# When a Palearctic bacterium meets a Nearctic insect vector: Genetic and ecological insights into the emergence of the grapevine Flavescence dorée epidemics in Europe

**DOI:** 10.1371/journal.ppat.1007967

**Published:** 2020-03-25

**Authors:** Sylvie Malembic-Maher, Delphine Desqué, Dima Khalil, Pascal Salar, Bernard Bergey, Jean-Luc Danet, Sybille Duret, Marie-Pierre Dubrana-Ourabah, Laure Beven, Ibolya Ember, Zoltan Acs, Michele Della Bartola, Alberto Materazzi, Luisa Filippin, Slobodan Krnjajic, Oliver Krstić, Ivo Toševski, Friederike Lang, Barbara Jarausch, Maria Kölber, Jelena Jović, Elisa Angelini, Nathalie Arricau-Bouvery, Michael Maixner, Xavier Foissac

**Affiliations:** 1 INRAE, Univ. Bordeaux, UMR BFP, Villenave d’Ornon, France; 2 Genlogs Biodiagnosztika Ltd, Budapest, Hungary; 3 Department of Agriculture, Food and Environment, University of Pisa, Pisa, Italy; 4 CREA Viticulture and Enology, Conegliano (TV) Italy; 5 Department of Plant Pests, Institute of Plant Protection and Environment, Zemun, Serbia; 6 CABI, Delémont, Switzerland; 7 JKI, Institute for Plant Protection in Fruit Crops and Viticulture, Siebeldingen, Germany; John Innes Centre, UK, UNITED KINGDOM

## Abstract

Flavescence dorée (FD) is a European quarantine grapevine disease transmitted by the Deltocephalinae leafhopper *Scaphoideus titanus*. Whereas this vector had been introduced from North America, the possible European origin of FD phytoplasma needed to be challenged and correlated with ecological and genetic drivers of FD emergence. For that purpose, a survey of genetic diversity of these phytoplasmas in grapevines, *S*. *titanus*, black alders, alder leafhoppers and clematis were conducted in five European countries. Out of 132 *map* genotypes, only 11 were associated to FD outbreaks, three were detected in clematis, whereas 127 were detected in alder trees, alder leafhoppers or in grapevines out of FD outbreaks. Most of the alder trees were found infected, including 8% with FD genotypes M6, M38 and M50, also present in alders neighboring FD-free vineyards and vineyard-free areas. The Macropsinae *Oncopsis alni* could transmit genotypes unable to achieve transmission by *S*. *titanus*, while the Deltocephalinae *Allygus* spp. and *Orientus ishidae* transmitted M38 and M50 that proved to be compatible with *S*. *titanus*. Variability of *vmp*A and *vmp*B adhesin-like genes clearly discriminated 3 genetic clusters. Cluster Vmp-I grouped genotypes only transmitted by *O*. *alni*, while clusters Vmp-II and -III grouped genotypes transmitted by Deltocephalinae leafhoppers. Interestingly, adhesin repeated domains evolved independently in cluster Vmp-I, whereas in clusters Vmp-II and–III showed recent duplications. Latex beads coated with various ratio of VmpA of clusters II and I, showed that cluster II VmpA promoted enhanced adhesion to the Deltocephalinae *Euscelidius variegatus* epithelial cells and were better retained in both *E*. *variegatus* and *S*. *titanus* midguts. Our data demonstrate that most FD phytoplasmas are endemic to European alders. Their emergence as grapevine epidemic pathogens appeared restricted to some genetic variants pre-existing in alders, whose compatibility to *S*. *titanus* correlates with different *vmp* gene sequences and VmpA binding properties.

## Author summary

Since the first outbreaks, Flavescence dorée epidemics had been associated to the introduction of the North American leafhopper vector *Scaphoideus titanus*. We hereby show that the associated phytoplasma originated from European alders and that epidemics in grapevine are restricted to some phytoplasma genetic variants pre-existing in this wild asymptomatic plant host. The compatibility of this phytoplasma to the introduced *S*. *titanus* insect vector resulted from the adaptation of phytoplasma variable membrane proteins Vmps to leafhoppers of the same subfamily living on alders. Vmps organization is similar to adhesion related proteins (ARP) and seems to allow the duplication of pre-adapted repeated domains. This suggests a key role of the Vmp adhesins in the life-style of phytoplasmas infecting woody hosts that rely on the adaptation to new insect vectors to expand their plant-host range.

## Introduction

Grapevine Flavescence dorée (FD) is a phytoplasma quarantine disease that emerged in the 1950s first in southwestern France and then in the vineyards of ten European countries [1, 2]. Its transmission in French and then Italian vineyards was quickly associated with the introduction of an American leafhopper, *Scaphoideus titanus* [3, 4]. In the late 1960s, the observation by electron microscopy of wall-less bacteria in the phloem-sap of FD affected grapevines oriented the etiological research towards new plant pathogenic agents, the mycoplasma-like organisms known nowadays as phytoplasmas [5, 6]. At that time, two phytoplasmoses already coexisted in the French and Italian vineyards, and the distinction between Bois noir, the first, and FD, the second, was first made on the basis of their different distribution in the vine plots and the ability or inability of *S*. *titanus* to propagate the disease [7]. Only the access to phytoplasma DNA made possible their phylogenetic classification as well as the taxonomic distinction of the different associated phytoplasmas [8]. The FD phytoplasmas (FDP) belong to taxonomic subgroups 16SrV-C and 16SrV-D [9–11]. If epidemiological surveys confirmed the diversity of FDP strains, their common property remained their ability to be transmitted by *S*. *titanus* [12, 13]. Before the 2000s, although it was commonly assumed that the pathogen was of North American origin, forest phytoplasmas such as those detected in black alders *Alnus glutinosa* in Germany and Italy turned out to be genetically closely related to FDP [14–16]. The 16SrV-C alder phytoplasmas, which did not always provoke symptoms in their plant host, were shown to be transmitted from alder to alder by *Oncopsis alni*, a monophagous leafhopper of subfamily Macropsinae, which can nevertheless transmit them to the vine [17–19]. These cases of grapevine yellows, called Palatinate grapevine yellows (PGY), were not transmissible by the *S*. *titanus* leafhopper [20]. In addition to alders, the climbing shrub *Clematis vitalba* was also found harboring 16SrV-C FDP, that could be transmitted to grapevine by the naturally infected planthopper *Dictyophara europaea* [21, 22]. At this stage it became clear that FDPs had a host range not restricted to grapevine and could possibly originate from Europe. It was first demonstrated using multi-locus sequence analysis that eight FDP genotypes clustered into three different genetic groups, which constituted with alder phytoplasmas a common monophyletic clade distinct from ‘*Candidatus* Phytoplasma ulmi’ (group 16SrV-A) and ‘*Ca*. P. rubi’ (group 16SrV-E), two other forest phytoplasmas also occurring in Europe [23, 24]. The objectives of the present study were to test the hypothesis of a European origin of FDPs and to identify genetic and ecological traits associated with their epidemic properties. A network of European laboratories set-up a plant and insect vector sampling in vineyards and wild surroundings in France, Germany, Italy, Hungary and Serbia. Phytoplasma strains were genetically characterized using the house-keeping gene *map*, whose diversity allowed to previously distinguish 17 different 16SrV-C and–D phytoplasma genotypes [23, 24]. Insect transmission assays were undertaken with naturally infected leafhoppers collected on German and French alders and the compatibility of these phytoplasma strains with *S*. *titanus* was tested. The correlation between the specificity of the strains to different insect vectors and the genetic diversity of *vmp*A and *vmp*B genes, two genes encoding adhesion related proteins, was finally evaluated as well as the ability of different VmpAs to interact with insect vectors.

## Results

### European black alders and alder leafhoppers commonly harbor diverse populations of FD and FD-related phytoplasmas

**Fig 1:**
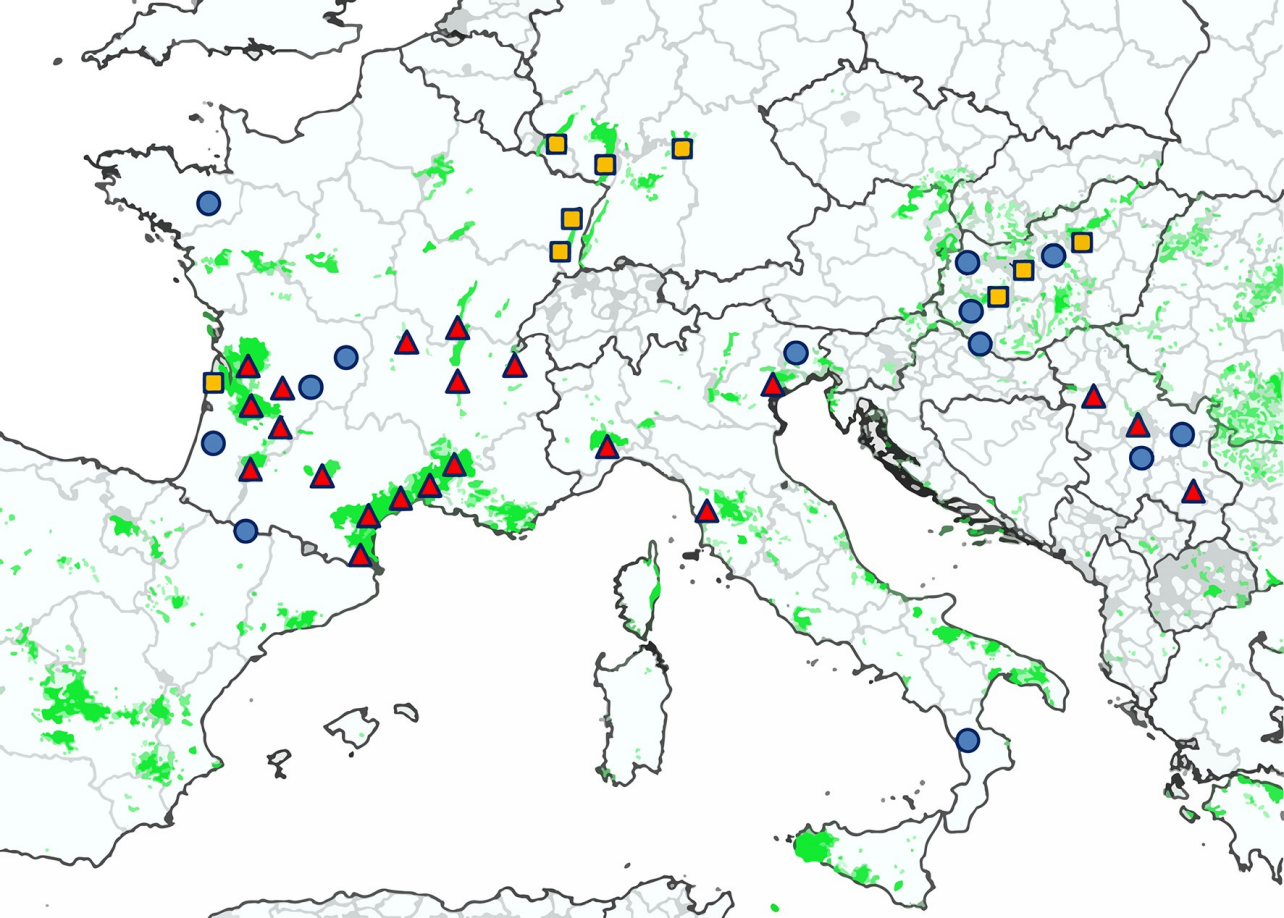
Location of samplings. Vineyards areas are appearing in green according to Corine Land Cover database CLC2012 release of the European Copernicus public program (https://www.copernicus.eu/en). Sampling sites were in non viticultural area (blue dots), in FD-free vineyards (yellow squares) and in FD-infected vineyards (red triangles) at the time of sampling. A detailed description of the sampled localities and the plant and insect sampled are given in Table 1 and S1 Table.

A sampling plan was drawn up to represent the diversity of ecological situations. It included wine-growing areas with or without FD and non-viticultural areas in five European countries, whether or not affected by FD (Fig 1 and Table 1). The sampling also included two areas north of the *S*. *titanus* distribution limit at that time. The phytoplasma group 16SrV-specific *map* gene nested-PCR assay was applied to all total nucleic acid samples. Eighty six percent of the 254 *Alnus glutinosa* trees sampled in the 5 countries were positive for 16SrV phytoplasmas, with an infection rate ranging from 77% to 100% between countries. Among the 46 *C*. *vitalba* collected in Hungary and France (Aquitaine and Alsace), only Hungarian samples were infected at 50%. Nearly 1500 *O*. *alni* (Macropsinae subfamily) were collected on alders in the different countries except Italy; the infection rate ranged from 19% to 22% between countries.

**Table 1:** Sampling plan and infection status of the samples.

Organism	Country	Administative regions or districts	Period	Nb samples	16SrV infection	Nb sequenced (*map*)^2^	Multiple sequences (*map*)^3^
***Alnus glutinosa***	France	Alsace, Aquitaine, Bretagne, Limousin, Midi-Pyrénées	2006–2010	104	77%	59	55%
	Germany	Bayern, Rheinland-Pfalz	1997–2010	40	80%	31	
	Hungary	Gyor, Heves, Pest, Somogy, Zala	2008–2010	38	92%	34	
	Italy	Basilicata, Friuli, Piemonte, Toscana, Veneto	2007–2010	45	100%	26	
	Serbia	Bor, Braničevo, Rasina, Šumadija	2007–2010	26	100%	26	
***Clematis vitalba***	France	Alsace, Aquitaine	2009–2010	24	0%	0	no
	Hungary	Fejer, Gyor, Heves, Zala	2009–2010	22	50%	11	
***Vitis vinifera***	France	Aquitaine, Alsace, Bourgogne, Midi-Pyrénées, PACA, Poitou-Charentes, Rhône-Alpes	2005–2011	37	Nd^1^	37	no
	Germany	Bayern, Rheinland-Pfalz	1995–2000	14	Nd^1^	14	
	Italy	Piemonte, Toscana, Valle d’Aosta, Veneto	2010	16	Nd^1^	16	
	Serbia	Beograd, South Bačka, Rasina, Nišava, Šumadija, Srem, Zaječar	2002–2006	14	Nd^1^	10	
***Scaphoideus titanus***	France	Aquitaine, Midi-Pyrénées	2005–2010	9	Nd^1^	8	no
	Serbia	Nišava, Srem	2010	4	Nd^1^	4	
***Oncopsis alni***	France	Alsace, Aquitaine, Bourgogne	2009–2015	568	22%	124	7%
	Germany	Rheinland-Pfalz	2009–2015	790	19%	108	
	Hungary	Somogy, Veszprem	2010	28	21%	2	
	Serbia	Bor	2010	68	20%	6	
***Allygus modestus* or *mixtus***	France	Alsace, Aquitaine, Bourgogne	2014–2015	71	40%	32	9%
	Germany	Rheinland-Pfalz	2014–2015	178	66%	108	
***Orientus ishidae***	France	Alsace, Aquitaine, Bourgogne	2014–2015	131	50%	66	4%
	Germany	Rheinland-Pfalz	2014–2015	23	61%	14	

1. Not done, samples were directly tested by nested-PCR *map*

2. Number of samples for which *map* gene was amplified and sequenced

3. Percentage of samples for which multiple sequences were observed for *map* gene

**Fig 2:**
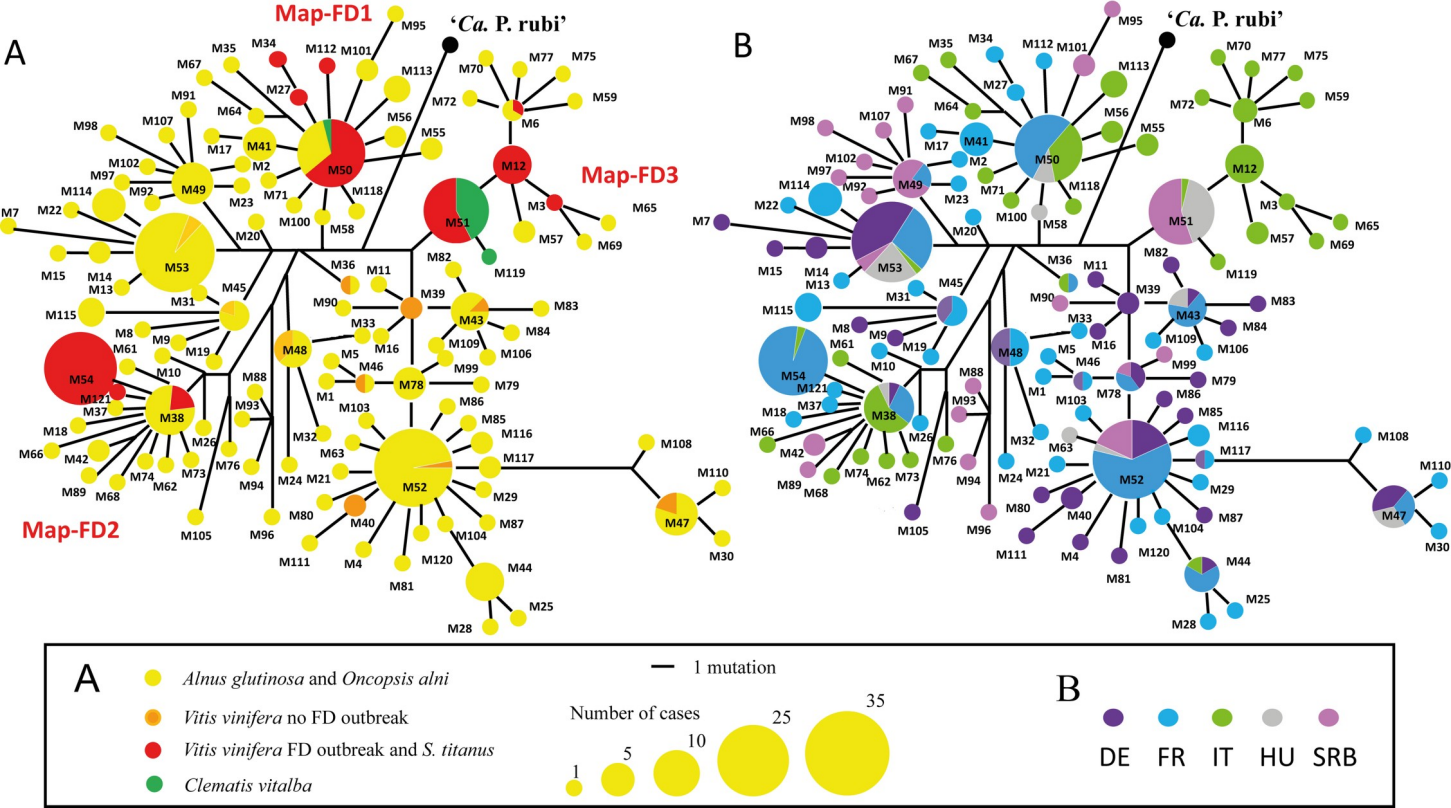
Genetic diversity of FD-related phytoplasmas in Europe. A. One of the trees constructed by parcimony analysis of the *map* gene nucleotide sequence (partial, 684bp) of the16SrV group phytoplasma isolates collected from *Alnus sp*. and *O*. *alni* (yellow), from *Vitis sp*. and *S*. *titanus* (orange and red) and from *Clematis vitalba* (green). Branch length is proportional to the number of inferred character state transformations and circle surface is proportional to the number of cases. B. Same tree representing the origin of phytoplasma isolates. France (F; light blue), Germany (G; purple), Hungary (H; grey), Italy (I; light green) and Serbia (SRB; pink).

The *map* amplicon was submitted to sequencing on a first set of 312 samples collected between 1995 and 2011 (Table 1 and S1 Table). For 55% of the alders, PCR products were composed of multiple *map* gene sequences, with 1 to 10 polymorphic positions, reflecting phytoplasma mixed infection by different genotypes. Single sequences of *map* gene were identified in all the other plants and in 93% of the positive leafhoppers. Resolution of genotype mixtures was carried out on 27 alder samples from the different countries by cloning and sequencing a minimum of four independent cloned amplicons produced with a proof-reading thermo-stable polymerase. Finally, 321 sequences were used for the phylogenetic analysis, including the sequences of 29 reference isolates (Fig 2). A total of 121 different sequences were identified and assigned to genotypes M1 to M121. Their corresponding accession numbers are indicated in S1 Table. The sequence of M60 displayed a T deletion at position 282 prematurely interrupting the coding frame that possibly occurred while cloning into *E*. *coli*. As M60 was found only once in multiple infection, it was excluded from the analysis. Two other sequences, namely M10 and M34, showed punctual mutations that resulted into alternative initiation codons, GTG at codon 1 for M10 and TTA at codon 7 for M34.

In grapevines and *S*. *titanus* collected in FD epidemic outbreaks, 11 genotypes were identified which belonged to the known genetic clusters Map-FD1, FD2 and FD3 [24] (Fig 2). The most abundant genotype, M54 from Map-FD2 cluster, corresponding to the French and Italian reference strains FD92 and FD-D, was present in all the French regions affected by FD, in Italy but not in Serbia. A two SNP variant of M54, M38 was detected for the first time in some single diseased grapevine plants in the French regions of Aquitaine, Rhône-Alpes and in the Italian region of Tuscany. A three SNP variant of M54, M121, was also newly detected in Rhône-Alpes. The genotype M50 from Map-FD1 cluster, corresponding to the French reference strains FD70 [24] and FD-CAM05 [25], was more abundant in outbreaks of South-West France, 36% of the FD cases, than in the other European countries surveyed and was also sporadically detected in Rhône-Alpes and Tuscany. Variants of M50, differing to M50 by 1 to 2 SNPs, namely genotypes M27, M34 and M112, were also identified in some isolated single cases in the French regions of Aquitaine, Midi-Pyrénées and Rhône-Alpes. The two prevalent Map-FD3 genotypes, M12 corresponding to the original North-eastern Italian strain FD-C [11, 14], and M51, were widely present in Italian and Serbian FD outbreaks respectively. The variants M3 and M6, respectively corresponding to the FD reference strains VI04-248-04 from Piemonte and VI04-TOSCANA1 from Tuscany [24] were obviously restricted to Italy. The infected grapevines collected in Germany and in the French Alsace, where *S*. *titanus* and FD were absent at the time of sampling, were infected by 10 different genotypes, which do not belong to clusters Map-FD1, FD2 and FD3. These genotypes, previously detected in German Palatinate and named PGY phytoplasmas [19], were often detected in alders (Fig 2).

Only 3 genotypes were identified in *C*. *vitalba*. The isolates collected from different viticultural and non-viticultural regions of Hungary were all M51, the only genotype found in Serbian FD outbreaks and more rarely in Italy (Map-FD3). The genotypes M119 and M50 were previously identified in Clematis in Italy [23].

A higher diversity was found in alder trees and *O*. *alni*, than in grapevine and *S*. *titanus*, as 115 genotypes were identified. In 55% of the alder samples, a mixture of different *map* genotypes could be detected. The most abundant alder genotypes were M52 and M53, which could be identified in all the countries but rarely in Italy. Interestingly, 8% of alder genotypes, namely M6, M50 and M38, were identical to grapevine genotypes (Map-FD1, 2 and 3) detected in FD outbreaks (Fig 1). In addition, these genotypes were detected in alder and/or in clematis of FD affected or FD-free viticulture areas as well as in non-viticulture areas. In details, M50 was found in alders in Italy and Hungary as well as in Clematis in Italy. M38 was found in alders in Italy, France and Germany; M6 was found in alders in Italy.

Sequencing of the 16S rDNA confirmed that all the isolates characterized belong to the 16SrV-C subgroup, except M54, which belong to 16SrV-D subgroup (S1 Table). Interestingly, M54 which is abundant in FD outbreaks in France and common in Italy, was never found in the surrounding alders, clematis or in alder leafhoppers.

**Fig 3:**
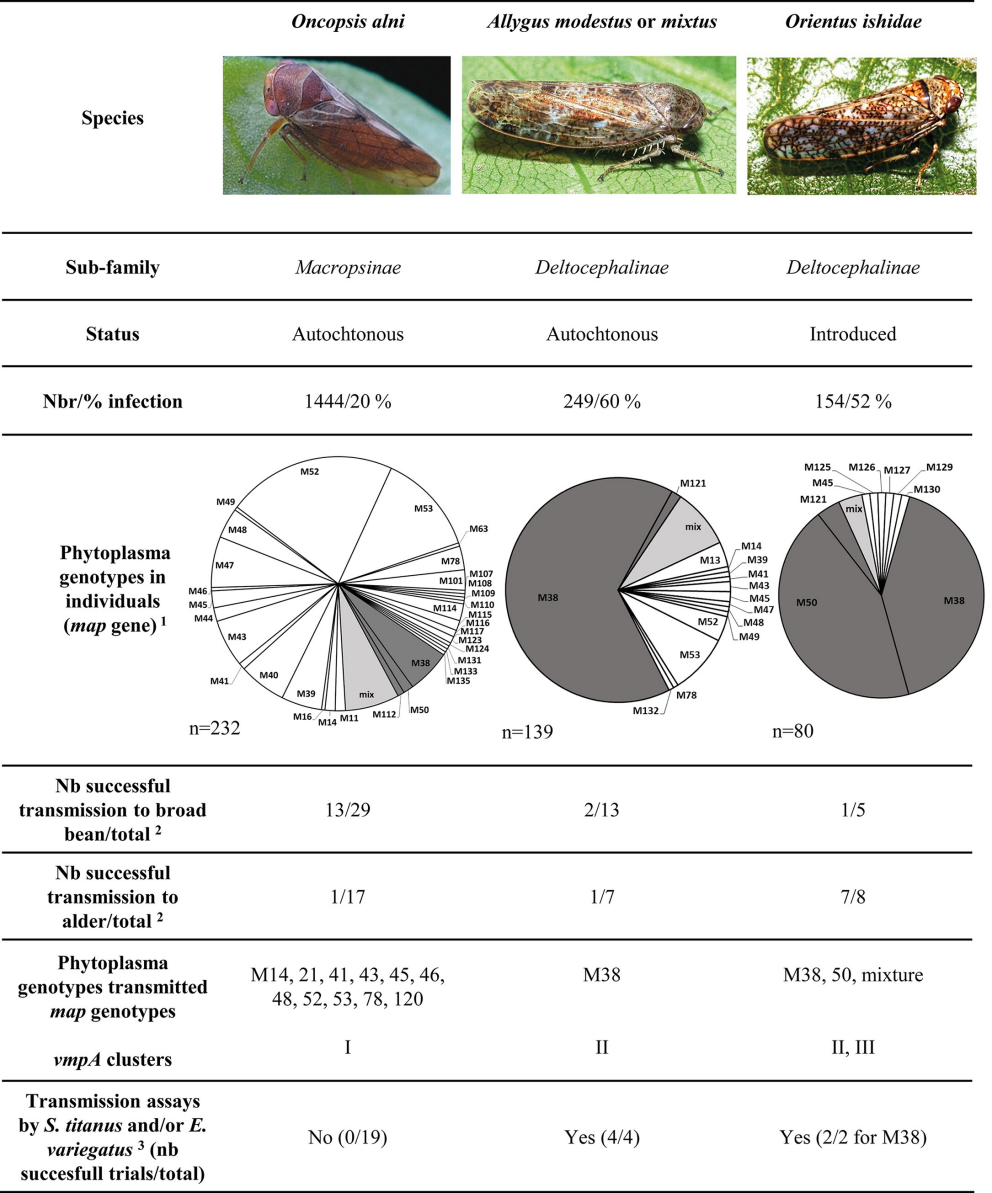
FD-related phytoplasma infection profile and transmission ability of leafhoppers collected on alder. Compatibility of transmission by *S*. *titanus* and *E*. *variegatus*. ^1^ Individuals infected by Map-FD1 and FD2 genotypes are represented in dark grey; genotypes not belonging to Map-FD clusters are in white; mixtures of genotypes are in light grey. ^2^ Natural transmission to one broad bean or alder plant with insects collected in the field on *A*. *glutinosa*. ^3^ A trial consists in an experimental acquisition on one infected broad bean followed by transmissions to 5–10 broad bean plants, either by *S*. *titanus* or *E*. *variegatus*, independently or consecutively (see S2 Table for details).

In 2014 and 2015, leafhopper collection on alders was extended to species of the Cicadellidae family other than *O*. *alni* in Aquitaine, Burgundy, Alsace (France) and Rhineland-Palatinate (Germany). Neither the 257 individuals from the Idiocerinae subfamily, *Idiocerus stigmaticalis*, *Idiocerus similis*, *Populicerus confusus* and *Tremulicerus vitreus*, nor the 10 *Macropsis* sp. individuals from the Macropsinae subfamily were infected. Deltocephalinae leafhopper populations of 249 *Allygus modestus/ mixtus* and 154 *O*. *ishidae* were collected and were found infected by 16SrV phytoplasmas in 60% and 52% of the individuals tested respectively. The *map* genotypes detected in each species, including *O*. *alni*, are indicated in Fig 3 and S1 Table. In contrast to alders, only 4% to 9% of the insects were infected with mixed populations of genotypes (Table 1). Supplementary new genotypes were identified and named from M123 up to M135 (accession numbers in S1 Table). Interestingly, whereas the prevalence of FD genotypes was only 8% in positive *O*. *alni*, it reached 67% in positive *Allygus* spp. and 89% in positive *O*. *ishidae*, which had been collected, for most of them, on the same alder trees (Fig 3, S1 Table). M38 was the major genotype detected in *Allygus* spp., while M38 and M50 had similar prevalence in *O*. *ishidae*.

### Alder Deltocephalinae leafhoppers naturally transmit FD phytoplasma genotypes able to fulfill transmission by *S*. *titanus*

Transmission assays were performed with *O*. *alni* collected between 2009 and 2015 and with *Allygus* spp. and *O*. *ishidae* collected in 2014–2015 in Alsace, Aquitaine, Burgundy and Rhineland-Palatinate. A total of 11 different map genotypes could be transmitted by *O*. *alni* to 13 broad beans over a total of 29 transmission assays (Fig 3 and S2 Table). These genotypes were distributed in different branches of the *map*-based phylogenetic tree and none of them corresponded to genotypes reported in FD outbreaks in Europe (Fig 2). Infected broad beans exhibited symptoms as severe as those exhibited by FD92-infected (M54, cluster Map-FD2) control broad beans and phytoplasma titers were similar with this control, ranging from 2.4x10^6^ to 3.8x10^7^ phytoplasma targets/μg total nucleic acids. Each infected broad bean served as feeding source for acquisitions by *E*. *variegatus* and/or *S*. *titanus* nymphs and after subsequent latency period, the insects were challenged for transmission to 5–10 broad bean plants per assay. None of the phytoplasma genotypes initially transmitted by *O*. *alni* could be transmitted, whereas transmission efficiencies ranged from 80 to 90% for the positive controls corresponding to strains FD92 and FD-PEY05 of genotypes M54 and strain FD-CAM05 of genotype M50. At the end of the transmission experiments, none of the insects were infected whereas between 80 and 95% of the insects were infected in positive controls.

Three transmission assays were successful with *Allygus* spp. over a total of 20 experiments. M38 (Map-FD2) was transmitted to two broad beans and one alder in agreement with the high prevalence of M38 in this leafhopper species (S2 Table, Fig 3). Transmission assays with *O*. *ishidae* led to one infected broad bean over 5 experiments and 6 infected alders over 8 experiments. The broad bean plant was symptomatic and infected with the M38 genotype (Map-FD2). The alders stayed non-symptomatic and were infected by M38, M50 (Map-FD1) or by a mixture of both genotypes, in agreement with their high prevalence in infected *O*. *ishidae*. Phytoplasma titers in the plants reached levels similar to the titers measured in control plants inoculated with FD92-infected *E*. *variegatus* (1.7x10^6^ to 1.3x10^7^ phytoplasma targets/μg total nucleic acids). When the M38-infected broad beans served as feeding source for *E*. *variegatus* and *S*. *titanus* nymphs, transmission rates were respectively 78% (14/18) and 60% (6/10), with respectively 30% and 83% infected insects at the end of the experiments. M50 transmission could not be assessed because it was only inoculated to alders, which are not a suitable feeding source for these leafhoppers. However, the strain FD-CAM05, our reference strain of genotype M50 that was initially transmitted by naturally infected *S*. *titanus* could be transmitted by *E*. *variegatus* in parallel experiments. Thus, the M38 genotype (cluster Map-FD2), naturally transmitted by the Deltocephalinae *Allygus* spp. and *O*. *ishidae*, was able to fulfill its transmission cycle in the Deltocephalinae leafhoppers *E*. *variegatus* and *S*. *titanus*.

### Gene sequences of phytoplasma surface proteins *vmp*A and *vmp*B correlate with *S*. *titanus* transmission

To investigate at genetic level the adaptation of the various phytoplasma genotypes to different insect vectors, the in silico-predicted proteome of FDP strain FD92 [26] had previously been searched for surface exposed proteins with organization in repeated domains reminiscent of adhesion related proteins of *Spiroplasma citri* [27]. Two genes, namely *vmp*A and *vmp*B had such organization and were therefore selected as candidates. Both genes encoded for proteins with N-terminal and C-terminal hydrophobic transmembrane alpha-helices surrounding a central hydrophilic region mainly composed of four repeated domains of 78 amino acids in VmpA and VmpB. This protein organization had previously been found in ‘*Ca*. Phytoplasma solani’ variable membrane protein 1 (Vmp1) [28]. At nucleotide level, *vmp*A and *vmp*B had no significant homologies but VmpA and VmpB proteins shared 39.4% identity at amino acid level. Both proteins could be serologically detected in phloem tissues of broad beans infected with FDP strain FD92 (S1 Fig).

**Fig 4:**
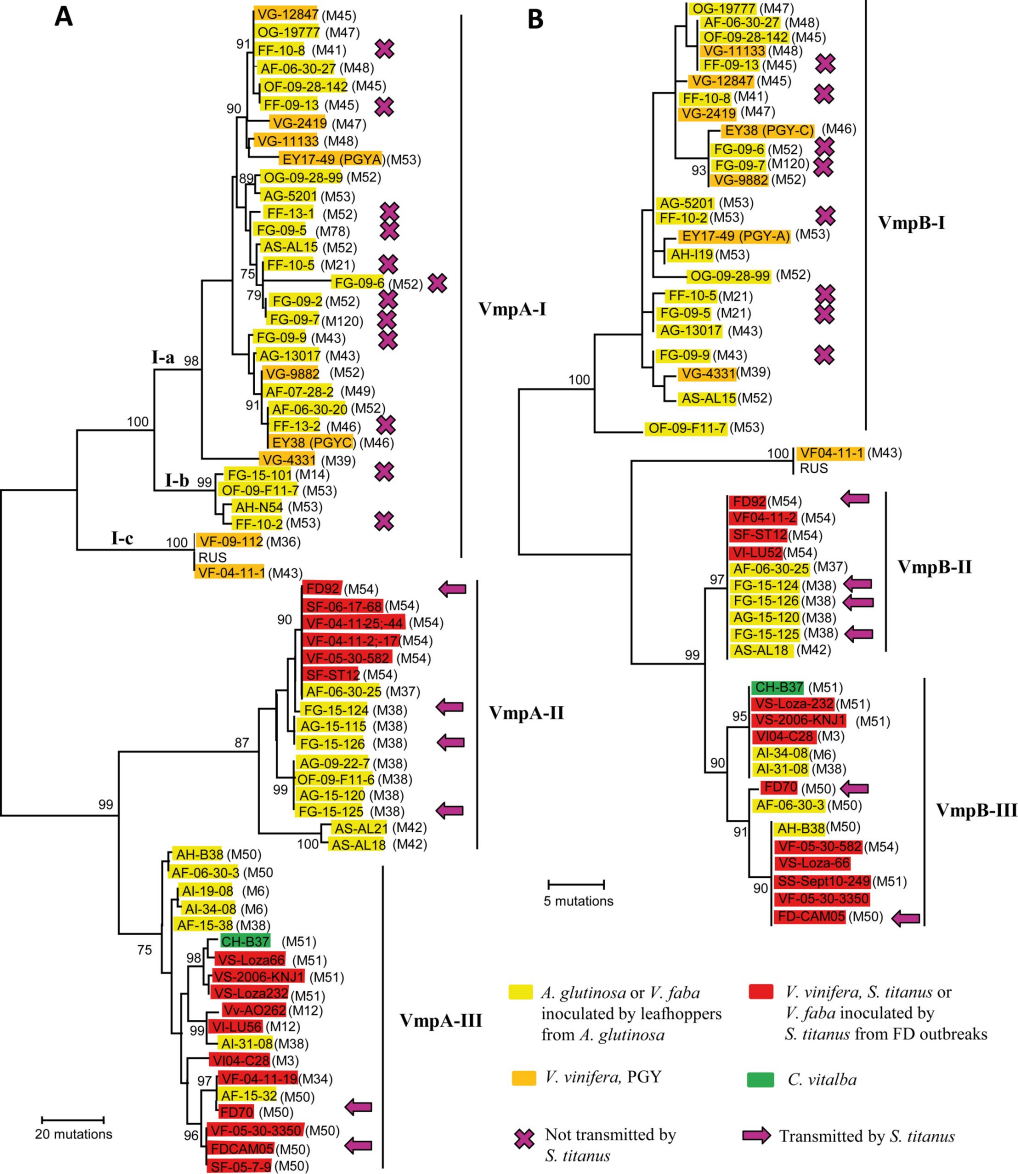
Sequence diversity of genes encoding Variable membrane proteins. One of the trees constructed by parsimony analysis of (A) *vmpA* gene and (B) *vmpB* gene nucleotide sequences. Except for phytoplasma reference strains, first letter of phytoplasma isolates indicate the biological origin, V for grapevine, A for alder, C for clematis, F for Faba bean issued from transmission assays and O for *Oncopsis alni*. Second letters of isolate names indicate the country of origin F for France, G for Germany, H for Hungary, I for Italy and S for Serbia. ClustalW was used to align vmp nucleotide sequences without prior alignment of protein sequences. The evolutionary history was inferred using the Maximum Parsimony method using the partial deletion option. The tree was obtained using the Subtree-Pruning-Regrafting (SPR) algorithm and was drawn to scale, with branch lengths calculated using the average pathway method in Mega5. Branch length are proportional to the number of inferred character state transformations and scale indicate the number of nucleotide changes. Bootstrap values for 500 replicates are indicated on branches when ≥ 75.

*Vmp*A and *vmp*B genes were sequenced for a panel of 70 and 50 isolates respectively, which represented a large diversity of *map* genotypes. Phylogenetic analyses revealed trees with similar topologies for *vmpA* and *vmpB* (Fig 4) but different from the topology of the tree based on the gene *map*. They were organized in three main genetic clusters with high bootstrap branching values, hereby named VmpA/B-I, II and III. Isolates from VmpA-I cluster were divided in three sub-clusters named a, b and c. None of the FD epidemic isolates from clusters Map-FD1, 2 and 3 were present in clusters VmpA/B -I, while they contained all PGY detected so far, various alder isolates of different map genotypes as well as the phytoplasma isolates transmitted by the alder *Macropsinae O*. *alni* which were not transmitted by the Deltocephalinae *S*. *titanus* and *E*. *variegatus* (Fig 3, Fig 4 and S2 Table). It is worth noticing that the ‘*Ca*. P. rubi’ strain RuS known to be transmitted by the Macropsinae leafhopper *Macropsis fuscula* [29] also had a *vmpA* gene of cluster I.

The VmpA/B cluster II included the FD epidemic isolates of the cluster Map-FD2 reported in Europe as well as the M38 isolates present in alders, transmitted by *Allygus* spp. and *O*. *ishidae* and experimentally transmitted by *S*. *titanus* and *E*. *variegatus*. Interestingly the alder isolates M37 and M42, which are M38 variants not yet reported in FD outbreaks, also grouped in cluster VmpA-II. Finally, the cluster III grouped the FD epidemic isolates, clematis and alder isolates from the Map-FD1 including M50 that could be transmitted by *O*. *ishidae*, and Map-FD3 clusters. Two alder isolates of genotypes M38 (cluster Map-FD2), namely AF15-38 from France and AI-31-08 from Italy, had *vmp* sequences of cluster III (Fig 3, Fig 4 and S2 Table). A M54 isolate, namely VF05-30-582, had a *vmp*A of cluster II but a *vmp*B of cluster III. This indicated that cluster VmpIII was represented in all Map-clusters, on the contrary to cluster II that was restricted to cluster Map-FD2.

### Vmp variability and domain duplication as possible adaptation trait to Deltocephalinae leafhopper vectors

**Fig 5:**
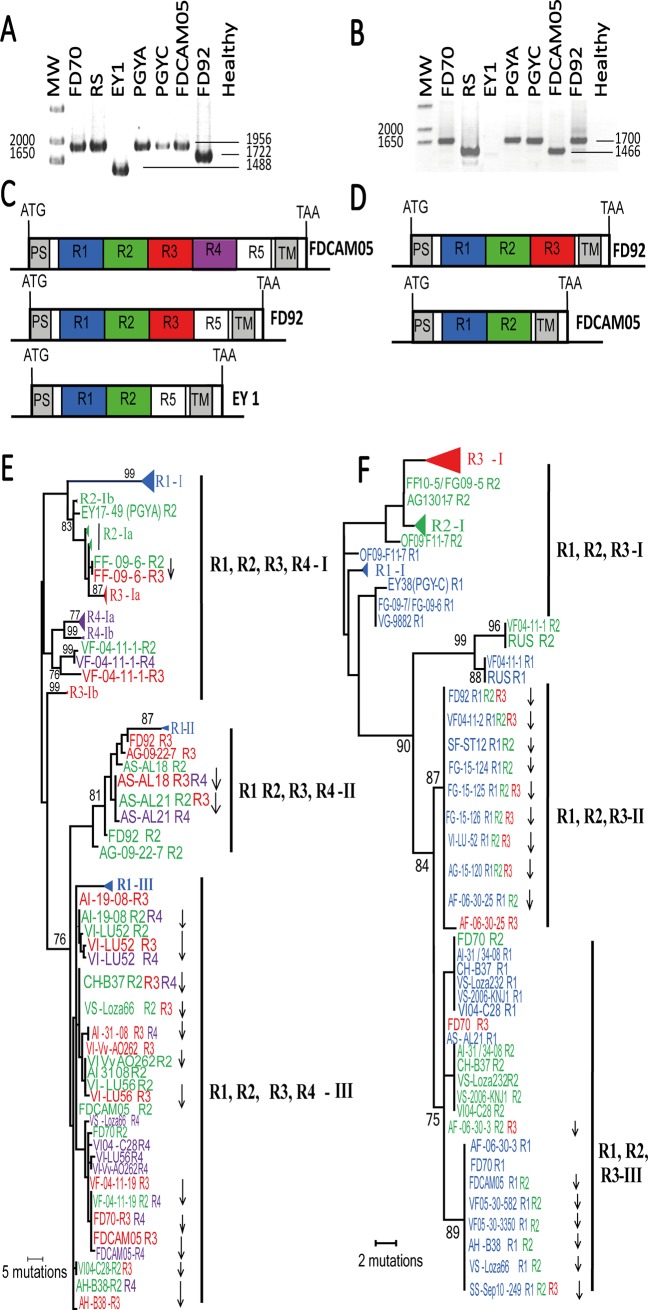
Number and sequence variation of the repeats constituting the *vmp* genes. (A) PCR amplification of *vmpA* and (B) *vmpB* genes from periwinkles infected with 16SrV phytoplasma refererence strains. Schematic representation of *vmpA* (C) and *vmpB* (D) genes with variable number of repeated sequences of 234 nt among 16SrV phytoplasma reference strains. PS is for signal peptide, R1 to R5 for repeated domains and TM for transmembrane segment. One of the trees constructed by parsimony analysis of the R1 to R4 nucleotide sequences (228 to 234 nt) of *vmpA* (E) and *vmpB* (F) genes. Branch length are proportional to the number of inferred character state transformations. Bootstrap values for 500 replicates are indicated on branches when ≥ 75. Down-pointing arrows indicate duplication events. Colours in phylogenetic trees indicate the position of the repeat as they appear in (C) and (D). Note that EY1 *vmp*B could be amplified using other primers described in S3 Table.

PCR amplifications revealed different sizes of *vmp*A and *vmp*B genes, which were confirmed by sequencing as being variations in the number of 234 bp repeated domains (Fig 5A to 5D). Sequences of VmpA-I and III clusters, including reference strains FD70, RuS, PGYA, PGYC and FDCAM-05, had four complete repeats named R1 to R4 (1956-bp long fragment) while VmpA-II sequences including the FD92 strain showed deletion of the R4 repeat (1722-bp long fragment). The Serbian isolates AS-AL18 and AS-AL21 were the only isolates lacking this deletion and the ‘*Ca*. P. ulmi’ reference strain EY1 only presented 2 repeats (1488-bp long fragment). Sequences of VmpB-I and II clusters, including reference strains PGYA, PGYC and FD92, showed three complete repeats (1703-bp long fragment). In this cluster, the isolates AS-AL21 and SF-ST12 constituted an exception with only one and two repeats respectively. Among sequences of the VmpB-III cluster, most of the isolates, including reference strain FD-CAM05, had 2 repeats (1469-bp long fragment), except FD70 and AF06-30-3 which showed 3 repeats.

Phylogenetic analyses were performed, based on the sequence of the repeats R1, 2, 3 and 4 for *vmp*A and R1, 2 and 3 for *vmp*B (Fig 5E and 5F). As expected, the phylogenetic trees presented the same topology as the trees based on the whole sequences, with the 3 clusters named I, II and III. The distribution of the repeats differed between each cluster. In cluster I, repeats were clustering by their positions in the gene, and were diversifying by accumulation of punctual mutations. On the opposite, in cluster II and III, *vmp*A repeats R2 to R4 and *vmp*B repeats R1 to R3 clustered with a within gene pattern, with the successive repeats being very similar, if not identical, suggesting they resulted from recent duplication events.

Tests of positive selection were performed on the repeat R1. The other repeats, which might be the result of duplication events, were not included in the analyses (S4 and S5 Tables). Most of the isolates from clusters II and III presented positive selection when compared with isolates of cluster I (p values < 0,05), whereas no positive selection could be evidenced between cluster II and III and within clusters.

### Enhanced adhesion of cluster II-VmpA to *E*. *variegatus* cell cultures and to midgut of *E*. *variegatus* and *S*. *titanus*

**Fig 6:**
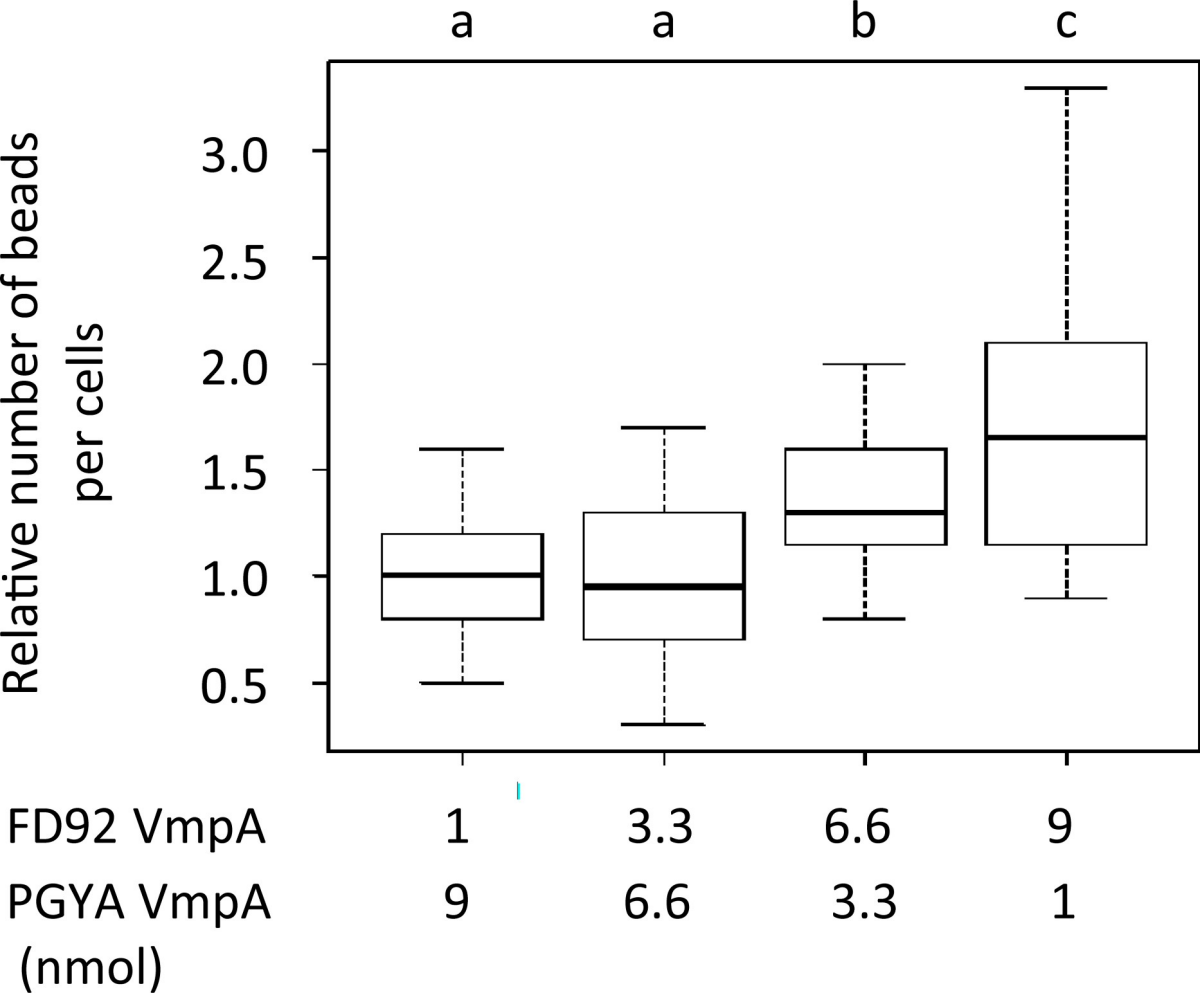
Adhesion of VmpA-His_6_-coated beads to the *Euscelidius variegatus* Euva-1 cells in culture. Fluorescent beads were coated with different amount of FD92 and PGYA VmpA-His_6_ (quantities of proteins indicated under the graph) before being in contact with insect cells in culture. The 100% relative correspond to the condition of cell adhesion with fluorescent beads coated with 1 mmol FD92 VmpA plus 9 nmol PGYA VmpA. Boxplot with different letters are significantly different under the AnovaModel of R.The obtained p-values with Kruskal-Wallis test of R were P = 0.0000041 for the VmpA-FD92:vmpA-PGYA protein ratio 9:1 vs 1:9 and P = 0.00012 for the VmpA-FD92:vmpA-PGYA protein ratio 6.6:3.3 vs 1:9.

To assess the specificity of adhesion of the protein VmpA of FD92 strain to the cells of *E*. *variegatus*, the Euva-1 cells in culture were incubated with fluorescent latex beads coated with different amounts of cluster II FD92 VmpA-His_6_ and cluster I PGYA VmpA-His_6_. Results showed that the higher the quantity of FD92 VmpA coating on beads, the higher the adhesion of beads to insect cells (Fig 6). Conversely, this was not observed for increasing quantity of PGYA VmpA. These results show that the adhesion of VmpA of the *S*. *titanus* transmissible strain FD92 to *E*. *variegatus* cells was more efficient than that of the VmpA of the *S*. *titanus* non-transmissible strain PGYA, when both VmpAs were present on the beads.

**Fig 7:**
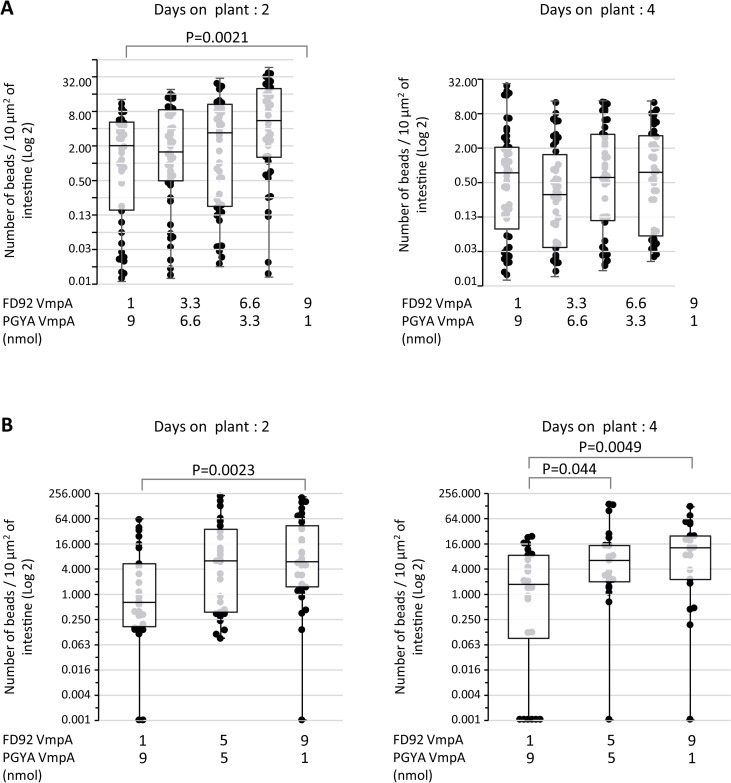
Number of FD92 VmpA-His_6_-coated latex beads in midgut of *Euscelidius variegatus* and *Scaphoideus titanus* after ingestion and fluorescence observations. Number of fluorescent beads coated with various concentrations of FD92 VmpA-His_6_ in *E*. *variegatus* middle midgut (A) or *S*. *titanus* middle midgut (B). After eating HEPES-sucrose containing the fluorescent beads, insects were maintained two and four days on broad bean before the intestine were dissected. A total of 43 to 60 insects per group were examined for *E*. *variegatus* (pool of 3 independent experiments) and 21 to 32 for *S*. *titanus* (pool of 2 independent experiments). P-value found with the Kruskal-Wallis test of R were indicated above de graph. Dots on the box-plots indicate Individual values.

After two days of bead ingestion by *E*. *variegatus* and two days on healthy broad beans, an increase of the beads retained in midgut was observed in relation with the quantity of FD92 VmpA coating on fluorescent beads, with a dose dependent manner (Fig 7A). This difference was statistically significant with the Student’s z-test between the conditions 1 nmol of FD92 VmpA-His6 proteins (average 7.2 ±10.8 and median 3.5 beads per 10 μm2 of medium midgut) and the condition 9 nmol of FD92 VmpA-His6 proteins (average 12.7 ±14.5 and median 4.4 beads per 10 μm2 of middle midgut). Similar results were obtained with *S*. *titanus* (Fig 7B). The difference of ingested beads was statistically significant with the Student’s z-test between the conditions 1 nmol of FD92 VmpA-His_6_ proteins (average 8.3 ±15.9 and median 0.6 beads per 10 μm^2^ of medium midgut) and the condition 9 nmol of FD92 VmpA-His_6_ proteins (average 35.5 ±56.4 and median 6 beads per 10 μm^2^ of middle midgut). After 4 days on plant, no difference was observed when *E*. *variegatus* ingested beads coated with VmpA whatever the condition was (Fig 7A), but significant differences were still observed when *S*. *titanus* ingested coated beads (Fig 7B), with average 5.4 ±7.3 and median 1.7 beads per 10 μm^2^ of medium midgut in the condition 1 nmol of FD92 VmpA-His_6_ proteins; and average 23.9 ±42.8 and median 6.4 beads per 10 μm^2^ of medium midgut in the condition 5 nmol of FD92 VmpA-His_6_ proteins; and average 23.8 ±30.8 and median 13 beads per 10 μm^2^ of medium midgut in the condition 9 nmol of FD92 VmpA-His6 proteins. Conversely, the increase of PGYA VmpA-His6 proteins did not improve the retention of the beads. These results show that VmpA of FD92 strain interacted more efficiently with the midgut perimicrovillar membrane of *E*. *variegatus* and *S*. *titanus* than the VmpA of PGYA.

## Discussion

### European alders are the original FDP reservoir

Since the first FD outbreaks in southwestern France, the epidemic propagation of the disease in grapevine was associated to a leafhopper of North American origin, *S*. *titanus* [3, 4]. This insect had been introduced in Europe, certainly in the late 19^th^ and early 20^th^ centuries, when American wild *Vitis* species resistant to phylloxera were imported to be used as rootstocks for the phylloxera-sensitive European *V*. *vinifera* [30]. The poor genetic diversification of *S*. *titanus* European populations recently provided evidence for a single major introduction from Northeast USA in Southwestern France [31]. Caudwell hypothesized that the FDP was as well introduced from North America [7, 32]. However, recent surveys reported that the grapevine yellows-associated phytoplasmas in USA and Canada are related to ‘*Ca*. Phytoplasma asteris’ and ‘*Ca*. Phytoplasma pruni’ [33–35], while 16SrV-C phytoplasmas were detected in wild plants such as *Apocynum cannabinum* in New York and *Parthenocissus quinquefolia* in Florida [24, 36, 37]. We previously demonstrated by MLSA that 16SrV-C and V-D phytoplasmas detected in European alders, clematis and grapevine had a common phylogenetic origin [23, 24]. We show in the present study that 16SrV-C phytoplasmas are endemic to the European common alders, which act most of the time as healthy reservoir for this bacterium. Altogether, 127 different genotypes of 16SrV-C phytoplasmas were detected in *A*. *glutinosa* and their feeding leafhoppers. Surveys in other regions of Germany, Serbia and North Macedonia identified 9 additional genotypes in alders [38, 39] and these genotypes were not detected in our surveys. The very high incidence in alders is leading in many cases to infection with multiple phytoplasma strains. The genetic diversity in alders, even when collected in FD-free and non-viticulture areas, was much higher than that found in grapevine. All the above observations confirm European alders as the original plant reservoir of this phytoplasma. *C*. *vitalba*, in which only three *map* genotypes could be detected in Serbia, Hungary and Italy might be considered a secondary epidemiological host for Italy, Balkans and possibly for other places in Europe.

### Possible multiple emergences from wild environment in Europe

If alders can easily be assumed as the original reservoir for 16SrV-C phytoplasmas, it is difficult to estimate the number of times these phytoplasmas successfully emerged into the grapevine-*S*. *titanus* epidemic cycle. The diversity of the neutral marker *map* in FD outbreaks possibly indicates at least 3 or 4 main independent FDP emergences, not contradicted by the observed diversity in *vmp* genes, including the less variable *vmp*B that shows 4 different sequences in clusters II and III. M50 (cluster Map-FD1) could have first locally emerged from European alders, as it was already present in 1970 in South western France (strain FD70). In early 2000s, M50 represented 15% of the FD cases in France [24], and in this study M50 was detected in FD outbreaks of Tuscany. M50 was also sporadically detected in Croatia [40], Switzerland [41] and in *C*. *vitalba* of Eastern Italian region of Veneto [23] and in north-western Italy [42], but was absent in the current alder and grapevine samples from Serbia. As shown by MLSA, the M50s detected in France, Veneto and Croatia were genetically different [23, 40], and we therefore cannot exclude multiple independent emergences of M50 from alders.

The second emergence could be that of M54 (cluster Map-FD2), that was isolated in 1992 in Southwestern France (strain FD92) [24]. In early 2000s, this strain represented 85% of the FD cases in France and was detected in Veneto [24]. We could detect M54 neither in Tuscany nor in Serbia. Although it was absent from Croatia before 2014, it had later on become frequent mainly due to the propagation of infected grapes of the cultivar Malvasia through nurseries [40]. M54 was absent in the alders tested so far, but the most closely related genotype M38, was regularly found during our surveys in French, Italian and at a lower extend in Hungarian and German alders. We could locally detect potential transfers of M38 from alders to grapevine in Eastern France, Southwestern France and Tuscany. Therefore, M38 transfer from alders could have preceded the emergence of M54 that most probably resulted from the subsequent diversification in grapevine of the ancestral M38 into M54. However, one cannot exclude that M54 diversified in alder before its transfer to grapevine.

Finally, because the genotypes detected in Balkan were nearly absent from Italy, and vice versa, multiple emergences may have occurred in the Map-FD3 cluster. The only genotype found in Serbian FD outbreaks, M51, was also recently reported as the only Map-FD3 genotype in Croatia [40] and the only genotype we detected in Hungarian *C*. *vitalba*. It was also the most prevalent genotype detected in *D*. *europaea* and *C*. *vitalba* collected in Serbia and Montenegro [43]. M51 was not detected in Italy during the present survey but was previously detected in Tuscany [23] and later on in Switzerland [44] and northwestern Italy [42]. On one hand, our data show evidence that M51 may have emerged from *C*. *vitalba* in Balkans and subsequently propagated westward to Italy where a secondary diversification into M12, M6 and M3 may have occurred possibly as a result of the presence of established *S*. *titanus* populations that migrated from France. On the other hand, as the M12, M6 and M3 genotypes were absent from Hungary and Serbia in our study and were previously shown to be also absent in Croatia [40], these genotypes may have emerged in Italy, either from alders or *C*. *vitalba*, independently of the M51 emergence in Balkans.

### New insights on the ecological cycle of FDP

**Fig 8:**
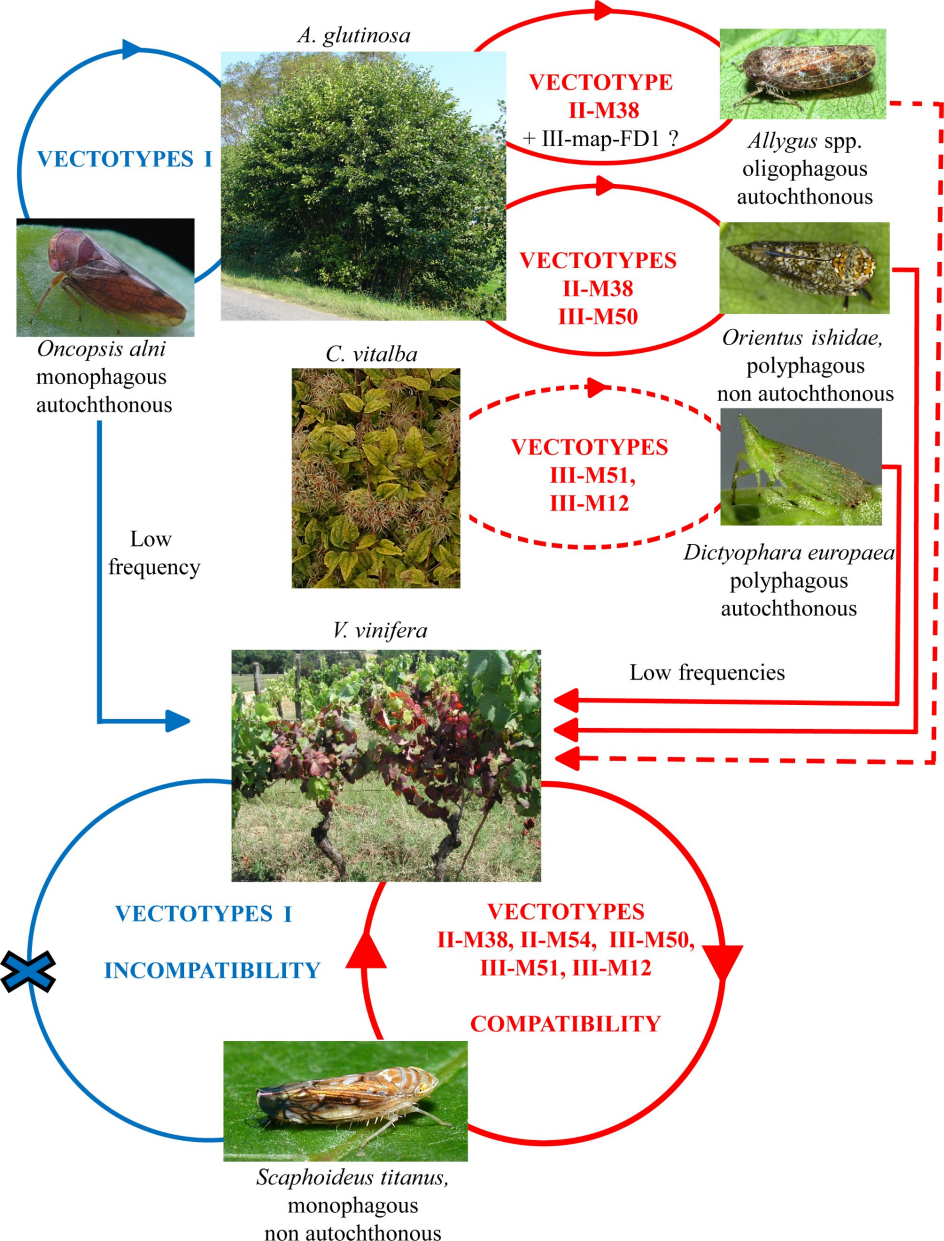
Ecological cycle of FD-related phytoplasmas as determined in this work and with previous literature. Vectotype I (Vmp-I): M43, M45, M47, M48, M49, M52, M53; Vectotype II (Vmp-II): M38, M54; Vectotype III-mapFD1 (Vmp-III): M50; Vectotype III-mapFD3 (Vmp-III): M51, M12 and possibly M3 and M6. The dotted line indicates possible transmission with no data available yet. The cross indicates that no transmission by *S*. *titanus* could be observed, neither naturally nor experimentally. Possible low transmission pathways of Vectotype III-mapFD1 (M50) by *Allygus* spp. or *O*. *ishidae* from *A*. *glutinosa* to *C*. *vitalba* are not indicated.

Recent works, including this study, unveiled the unpreceded complexity of FDP ecological cycle (Fig 8). Among the many 16SrV-C genotypes found in alders, the genotypes M38, M50, M52, M53 and at a lower extend genotypes M43, M47, M48, M49 were found abundant in at least two countries. Despite this ecological success, our data suggest that only M50 and M38 escaped from alders and propagated in the *V*. *vinifera-S*. *titanus* pathosystem. Based on our hypothesis that alder-feeding leafhoppers, other than the monophagous *O*. *alni* (subfamily Macropsinae) act as vectors for this limited part of the European16SrV-C phytoplasma population, we focused our attention on Deltocephalinae leafhoppers, the subfamily to which *S*. *titanus* belongs, because this subfamily was shown to transmit FDP upon microinjection and artificial feeding bioassays [45]. Our transmission assays confirmed that *O*. *alni* only transmitted phytoplasma genotypes never detected in FD outbreaks, whilst our assays newly demonstrated that Deltocephalinae *Allygus* spp. and *O*. *ishidae* naturally acquire and transmit M38 and M38/M50 respectively. We also show herein that *S*. *titanus* transmits M50 naturally and M38 experimentally. At the time of FD emergence native leafhopper species such as *A*. *modestus* and *A*. *mixtus* [46], could have been responsible for the transfer of M38 to grapevine, even though it remains to be experimentally reproduced. *O*. *ishidae*, the polyphagous mosaic leafhopper, originates from Eastern Palearctic [47] and was only reported in the European vineyards and their vicinity in the year 2000 [48–53]. It was shown to be infected with FDP genotypes also detected in FD outbreaks [41, 51, 52, 54] and to be able to transmit FDP to grapevine after experimentally forced acquisition [48].

For the management of FD epidemics, it is important to estimate the frequency of transfer of FDP from wild alternative hosts to surrounding vineyards. Ongoing surveys of the vineyards surrounding the alders on which infected *Allygus* spp. and *O*. *ishidae* were collected, point out a limited rate of FDP transfer to grapevine. For instance, only two cases of M38 and M50 were detected by plant-protection services over 6 years of exhaustive prospections at our insect sampling site in Burgundy. In a local study case in Southern Switzerland, *O*. *ishidae* was recently found infected with M50 (Map-FD1), M54 (Map-FD2) and M51 (Map-FD3), all being also detected in the hazelnut *Corylus avellana*, suggesting that *O*. *ishidae* could have locally transmitted FDP to a new host [44]. The impact on FD epidemiology of this new ecological cycle, especially the rate of transfer from FDP-infected hazelnuts to the surrounding vineyards is still unknown.

Regarding the epidemiological cycle on *C*. *vitalba*, it is currently difficult to explain the high FDP infection rate of *C*. *vitalba* we measured in Italy, Serbia and Hungary without speculating the existence of another FDP vector responsible for the FDP spread in *C*. *vitalba* natural populations. Two arguments are in favor of this speculation. First, the European lantern fly *D*. *europaea*, was previously described as transfer vector from *C*. *vitalba* to grapevine after experimental acquisition on FDP*-*infected *C*. *vitalba*, but has not so far been demonstrated to transmit FDP back to *C*. *vitalba* [22]. Second, *D*. *europaea* populations can show very low level of FDP infection despite high level of FDP infection rate of local *C*. *vitalba* populations [43].

Our study adds additional information on population genetics of FDP and related phytoplasma of 16SrV-C and -D. It especially brings explicative genetic markers that lead us to define the new concept of Vectotypes. The vectotype concept is based on the ability of phytoplasma strains carrying a given non- neutral marker, here a given adhesin Vmp-type, to achieve their complete transmission cycle in a given insect or group of insects. Hence, we propose to classify M38 as Vectotype II, like M54, as they also belong both to the same *vmp* genetic cluster II. In a similar way, M50 and M51 should be both classified as Vectotype III (Fig 8) as they share *vmp* type III and can also achieve their complete transmission cycle though *S*. *titanus*. The Ald and PGY phytoplasma strains tested so far belong to the Vectotype I and are incompatible with *S*. *titanus* transmission. The denomination FDP can therefore be restricted to Vectotypes II and III, finally reconciling FDP molecular definition and its initial biological definition restricted to 16SrV-C and V-D phytoplasmas transmitted by *S*. *titanus*.

### Genetic traits of FDP compatibility to *S*. *titanus* as new useful clues for FD management

To achieve their complex life cycle in the insect vectors, phytoplasmas, like plant pathogenic spiroplasmas, must colonize the epithelium of the insect midgut and ultimately the salivary glands, to be inoculated to plant sap through infected saliva [55–57]. The first steps of this cycle are the adhesion to the epithelial cells of the insect vector, internalization, multiplication, intracellular trafficking and crossing the basal membrane and lamina of midgut epithelium to reach other tissues. The initial adhesion process is quite well deciphered for *Spiroplasma citri*. Spiralin, the main *S*. *citri* surface lipoprotein, acts as a lectin that binds insect vector glycoproteins [58], relocalizes at the adhesion contact point, and is required for efficient transmission [59, 60]. In addition, the *S*. *citri* adhesion related proteins (Scarps) also participate to the adhesion and all had repeated domains of 38–40 amino acids, that triggers entry of *S*. *citri* into cultured cells of the insect vector *Circulifer haematoceps* [27, 61, 62]. In phytoplasmas, two types of surface proteins, Amp and Vmp, have confirmed role in the interaction with insect vector. The ‘*Ca*. P. asteris’ Amp binds actin microfilaments of the insect vector, possibly promoting intracellular trafficking [63]. *Amp* gene and its orthologue in ‘*Ca*. P. solani’ are both submitted to strong positive diversifying selection, suggesting adaptation processes [64, 65]. Regarding primary adhesion of phytoplasmas to the insect cells, we initially looked for genes under positive selection and possessing repeated domains as it was found in Scarps. A gene fitting this criteria, *vmp*1, was initially found in ‘*Ca*. P. solani’, but no functional studies could be undertaken in the absence of insect vector colonies [28]. In the case of VmpA, an FDP surface protein analogous to Vmp1, we could demonstrate that VmpA acts as an adhesin able to bind to insect vector cells in culture and to its midgut perimicrovillar membrane [66, 67]. We show here that sequence variations of *vmp*A and *vmp*B genes correlate with transmission by insect vectors of different leafhopper subfamilies. Only the phytoplasma variants carrying *vmp* genes of genetic clusters II and III are transmitted by *S*. *titanus* and provoke FD outbreaks. The 16SrV-C strains with *vmp* genes of cluster II and III are pre-existing in alders and we showed that they are propagated by *Allygus* spp. and *O*. *ishidae*. Native alder leafhoppers such as *Allygus* spp. could have pre-adapted these forms of Vmps to the Deltocephalinae leafhoppers in general.

In the absence of genetic engineering of the uncultivated phytoplasmas, we previously used protein-coated latex beads or recombinant spiroplasmas to mimic phytoplasma surface, and demonstrate that VmpA acts as an adhesin binding to the cells of *E*. *variegatus* [66]. Here we found that latex beads had enhanced adhesion to *E*. *variegatus* cells in culture and were better retained in *E*. *variegatus* and *S*. *titanus* midguts, when coated with increasing ratio of FD92 VmpA (cluster II) over PGYA VmpA (cluster I). This suggests that VmpA of strain FD92 could be better adapted to interact with Deltocephalinae leafhopper midgut than VmpA of strain PGYA, which is naturally transmitted by the Macropsinae *O*. *alni*. Mutations in bacterial adhesins can cause changes in tissue tropism or host range, linked to variation in affinity [68, 69]. The variability of VmpA sequence and its congruence with insect vector classification suggest that insect receptors to VmpA could differ between leafhopper sub-families, and therefore explain the change in phytoplasma-vector specificity. We however have no evidence that VmpA of strain PGYA and that of cluster I in general show enhanced adhesion to *O*. *alni*. The expansion of protein domains repeat is common in proteins with various binding properties [70]. Duplication but also deletion can arise from *recA*-independent replication slippage of direct-repeat sequences or *recA* dependent recombination mechanisms [71, 72]. The duplication of *vmp* repeated domains was observed for FDP strains of clusters II and III transmitted by Deltocephalinae leafhoppers. Deletion of *vmp* domains, as we could observe in *vmp*B of the Serbian alder isolate AS-AL21 that possesses only one domain, could help resetting the *vmp* gene, prior to further diversification and duplication. These two molecular phenomena seem to take place in both *vmp* genes but are likely more recent in *vmp*B than in *vmp*A, in which VmpA domain 1 appeared differentiated enough to escape duplication. In GNA-related lectins, generation of multispecificity is generated through domain duplication and divergent evolution [73]. The identification of the Vmps receptors and the nature of their interaction with Vmps will help to measure the possible impact of domain duplication on Vmp adhesion efficiency and specificity.

Last but not least, as phytoplasma strains with *vmps* of cluster I are unlikely to provoke outbreaks through *S*. *titanus* transmission, it is not necessary to spray insecticide in vineyards where such phytoplasma strains are detected. Such experimental management is currently on-going in France, in regions where FD is absent or has recently emerged. As a first applied outcome, *vmp* typing should be helpful to reduce insecticide use in the management of FD.

Finally, the use of FD model is of general interest to reconstruct and trace the process of adaptation to a new vector as main risk factor promoting emergence of vector-borne diseases into agricultural systems.

## Methods

### Source plants, leafhoppers and phytoplasma reference strains

*Vicia faba* cultivar Agua dulce and *A*. *glutinosa* were grown from seed and *V*. *vinifera* (Cabernet Sauvignon) from hot water treated certified canes. The experimental vector of FD phytoplasma, *Euscelidius variegatus*, was reared on a combination of *V*. *faba* and *Avena sativa* oats. *S*. *titanus* was grown in small plastic incubators from eggs of two years old *V*. *vinifera* canes collected in FD free vineyards from Burgundy which were previously recorded with high populations. Hatchings and larvae development were performed as described in Eveillard et al. 2016 [74]. Phytoplasma reference strains and isolates used in the study have previously been described and are listed in S1 Table [23, 24, 75]. They were kept as DNA extracts or maintained in *Catharanthus roseus* by grafting. Phytoplasma strains FD92, FD-PEY05 and FD-CAM05 were transmitted to broad bean by using *S*. *titanus* leafhoppers collected in FD-affected vineyards of South-west France [25]. The strains have since been maintained by serial transmission from broad bean to broad bean using *E*. *variegatus* as an alternative leafhopper vector. All plants and insects were manipulated in confinement greenhouse.

### Plants and leafhoppers sampling

The majority of plant and insect samples were collected between 2005 and 2015 from the end of June to the end of October in Hungary, France, Germany, Italy and Republic of Serbia in the surroundings of FD-infected vineyards, known FD-free vineyards and also in non-viticultural areas. Sampling plan is summarized in Fig 1 and Table 1. Name, geographical origin and genotyping data of the samples are listed in S1 Table. Leaves with petioles were sampled from *C*. *vitalba* exhibiting yellows as described in Filippin et al. 2009 [22]. Single canes were cut from *A*. *glutinosa* without typical symptoms. Various leafhoppers from the Cicadellidae family were collected by beating on alder trees. Leafhoppers were immediately sorted by species or genus for transmission assays and/or transferred in EtOH 70% for further analysis. They were identified in accordance with common handbooks for taxonomy of leafhoppers [46, 76]. Infected samples of *V*. *vinifera* and *S*. *titanus* leafhoppers from various geographic origins and representative of the FD and PGY phytoplasma genetic diversity were also provided by the laboratories involved in the study. Finally, 25 previously described reference samples [23, 24, 75] were included in the sample’s set. All of them are listed in S1 Table.

### Phytoplasma detection and quantification

Total nucleic acids (TNA) from samples was extracted with the CTAB method as described in Maixner et al. 1995 [77]. Insects were ground individually in 250 μl CTAB extraction buffer and the final pellet was resuspended in 40 μl TE 1X. Half gram of petioles from symptomatic leaves or scratched liber from canes were ground in 3 ml CTAB buffer and the final pellet was resuspended in 100 μl TE 1X. TNA from healthy leafhoppers or plants issued from greenhouse was used as a negative control for each extraction series. Phytoplasmas of the 16SrV group were detected on total TNA extracts by nested-PCR either on *sec*Y (FD9) or *sec*Y-*map* locus [24, 78]. Phytoplasma titers were determined as the number of phytoplasma targets/μg total nucleic acids as previously published [74].

### Amplification and sequencing of genetic markers

The 16S rRNA gene was amplified by nested-PCR and sequenced using the P1, P7 and R16F2n primers [79, 80]. The secY-map locus (*map* gene) was amplified by nested PCR and sequenced as previously described [24]. If sequencing revealed a mixture of different *sec*Y*-map* PCR products, the locus was amplified with the high-fidelity DNA polymerase “DyNAzyme EXT DNA” (Finnzyme) by 25 cycles of the first PCR as described in Arnaud et al. 2007 [24]. PCR products were cloned in the pGEMT-Easy plasmid (Promega) as described by the manufacturer and 4 clones were submitted to sequencing. The *vmp*A and *vmp*B genes (accession numbers LN680870 and LR585965 respectively) were isolated and characterized from the whole genome sequencing and annotation of the FD92 phytoplasma strain [26]. Primers used for the nested-PCR amplification and sequencing of *vmpA* and *vmpB* were defined from the sequences of 16SrV group reference strains EY1, EY17-49 (PGY-A), EY38 (PGY-C), FD-CAM05, FD70, FD92 and RuS. Primer sequences and PCR conditions are detailed in S3 Table. Sequencing reactions were performed by Beckman-Coulter Genomics (Takeley, UK) by Sanger sequencing on Applied Biosystem 3730XL instrument with primers detailed in S3 Table.

### Genetic variability analyses

The raw sequence chromatograms were assembled and edited using Phred, Phrap, Consed or Gap4 [81]. Multiple sequences alignments were performed using the ClustalW program [82]. Phylogenetic reconstructions using maximum parsimony were performed by MEGA5 [83], with randomized bootstrapping evaluation of branching validity for *vmpA* and *vmpB*. Sequences were deposited at EMBL and accession numbers are listed in S1 Table. Selection pressure was evaluated using MEGA5 software by codon-based Fisher’s exact test of selection with the modified Nei-Gojobori method and a transition/transversion rate (R) calculated at 2. All sequences were deposited in the European Nucleotide Archive under the following accession numbers: LT221896 to LT222016 for *map* genotypes M1 to M121, LR585147 to LR585207 for *vmp*A, *vmp*B and additional *map* sequences M123 to M135.

### Detection of VmpA and VmpB expression by epifluorescence and confocal-microscopy

Polyclonal antisera have been raised in rabbit by COVALAB (Villeurbanne, France) against the hydrophilic central domain of VmpA and VmpB expressed and purified from *E*. *coli* as previously reported [66]. Fresh stems of healthy and FDP strain FD92-infected broad bean *Vicia faba* cv. Agua dulce were cut in sections of 10 μm with a freezing microtome. The sections were dried 30 min at 65°C, saturated 1h at room temperature in PBS 1X 1% BSA and incubated 1h at room temperature with the rabbit anti-VmpA or anti-VmpB polyclonal antibodies diluted 500 times in PBS 1X. Sections were rinsed 3 times in PBS and incubated in the dark with 10,000-fold dilutions of anti-rabbit sheep-IgGs labeled with fluorescein isothiocyanate for epifluorescence microcopy or labelled with Alexa 488 for confocal microscopy. Sections were rinsed 3 times in PBS 1X buffer containing 0.01% Evans blue (S1A1 and S1A2 Fig) or in PBS 1X buffer (S1B1 and S1B2 Fig). Sections were covered by a mounting solution and observed in a Zeiss III RS fluorescent microscope with the filter combination PB 455/490 FT 510 LP520 (S1A1 and S1A2 Fig) or were mounted in the anti-fading ProLong Gold Reagent (Invitrogen) and immunofluorescent samples were imaged using a TCS SP2 upright Leica confocal laser scanning microscope (CLSM).

### Transmission experiments

Leafhoppers from the Cicadellidae family were collected on infected alder trees from the beginning of June until the end of July. They were immediately sorted by genus or species and placed by groups of 10 to 40 on healthy *V*. *faba* or *A*. *glutinosa* plants until death. The dead insects were collected every day and stored frozen for further testing. Plants were incubated in a confined greenhouse for up to 10 weeks at 25°C constant and regularly tested for symptoms and for the presence of 16SrV phytoplasmas. If necessary, *A*. *glutinosa* were subjected to a dormancy period in a cold greenhouse and tested again after burst the following spring.

To evaluate the transmissibility of phytoplasma strains originating from alders with *S*. *titanus* and *E*. *variegatus* natural and experimental vectors, phytoplasma-positive *V*. *faba* exhibiting severe symptoms were incubated one week with 120 fourth or fifth instar larvae of *S*. *titanus* followed by one week with 120 4–5 instar larvae of *E*. *variegatus* for phytoplasma acquisition. *S*. *titanus* and *E*. *variegatus* were transferred on healthy *V*. *vinifera* and *V*. *faba* respectively, for a latency period of 4 weeks and then placed for transmission by groups of 10 on new young *V*. *faba* seedlings until death. Positive control transmission assays by *S*. *titanus* were performed in high confinement greenhouse with the same lots of insects placed for acquisition on FD92 and FD-PEY05 infected *V*. *faba* as positive controls. Positive control acquisition and transmission assays by *E*. *variegatus* were also routinely performed on broad beans with strains FD92, FD-PEY05 and FD-CAM05. Dead insects were collected and tested for 16SrV phytoplasma presence as described above. Plants were incubated up to 12 weeks and regularly tested for 16SrV phytoplasma presence and checked for symptoms. Alders were also monitored and tested after a winter-dormancy period. Phytoplasma were quantified in infected plants as described in Salar et al. 2013 [75] or Eveillard et al. 2016 [74] and genotyping of phytoplasma strains was performed as described above.

### Euva-1 adhesion assays

The yellow-green fluorescent and amine-modified beads (4x10^9^ beads, 1 μm) (Invitrogene) were covalently coated with 10 nmol of a mixture of recombinant VmpA-His_6_ of FD92 and PGYA phytoplasma strains, according to the supplier’s instructions and as previously described [66]. Coating of the beads was verified by immunofluorescence and the Bradford procedure.

Adhesion assays of VmpA-His_6_-coated beads were performed as previously described [59, 66]. Briefly, Euva-1 cells cultivated on coverslips in 24-well plates were incubated with 2x10^6^ coated latex beads in 500 μl culture medium for 1 h at 25°C. After three washes, the cells were fixed with 4% paraformaldehyde, and the cell nuclei were stained with 1 μgml^−1^ DAPI (SIGMA) for 5 min. The samples were mounted in the anti-fading ProLong Gold Reagent (Thermo Fisher Scientific), and immunofluorescent samples were analyzed with a fluorescence microscope (Nikon Eclipse E800) at 40× magnification. Each experiment was repeated three times independently. For each experiment, 20 fields with approximately 30 cells per field were observed randomly. Counting of beads per cell, performed with the free software package ImageJ (http://imagej.nih.gov/ij/), and calculation of the relative number of adherent beads per cells were performed as previously described [66].

### Ingestion assays

Ingestion assays were performed as previously described [66]. Briefly, 3 young adults of *E*. *variegatus* were introduced into a tube in which the cap was filled with 500 μl HEPES 8 mMsucrose 280 mM pH 7.8 solution containing 10^5^ coated beads. After two days at room temperature, 25 to 30 insects were transferred into a cage containing 2 broad beans. Two and four days later, about 20 insects were dissected and midguts were stained with 1 μgml^-1^ DAPI after fixation with 4% paraformaldehyde in PBS containing 0.1% Triton X-100. The organs were mounted and middle midguts were observed as the Euva-1 cells were. For each experiment, approximately 15 midguts were observed per condition, and the experimentation was repeated three times. Counting of beads per midgut and the determination of the area of midguts were performed with the free software package ImageJ (http://imagej.nih.gov/ij/). Ingestion assays were also performed with *S*. *titanus* using 2 young adults per tube and a HEPES 4mM-sucrose 140 mM pH 7.8 solution.

### Statistical analysis

To evaluate the statistical significance of the values, similarities of deviations between independent experiments were first checked with the F-test. The normal distribution was tested with the Normality test (Shapiro-Wilk test) of R (Rcmdr package). Then, for statistical evaluations, when a P-value >0.05 was obtained using the Normality test (Shapiro-Wilk test) the AnovaModel of R was used, and when a P-value <0.05 was obtained using the Normality test (Shapiro-Wilk test) the Kruskal-Wallis test of R was applied.

## Supporting information

S1 Fig Serological detection of VmpA and VmpB in FD92 FDP-infected *Vicia faba*. A: anti-VmpA monoclonal antibody detected with FITC labelled anti-mouse secondary antibodies in epifluorescent microcoscopy. B: anti-VmpB polyclonal antibodies detected with Alexa 488 labelled anti-mouse secondary antibodies in confocal microscopy. A1 and B1: healthy faba beans, A2 and B2: faba beans infected with FDP strain FD92. Xyl indicate xylem tissues and Phl indicate phloem tissues.
(TIF)

S1 Table Origin and genotype of phytoplasma isolates. (XLS)

S2 Table Transmission trials of FD-related phytoplasmas by *O*. *alni*, *Allygus* spp. and *O*. *ishidae* collected on alder and subsequent transmission by *E*. *variegatus* and *S*. *titanus*. (DOCX)

S3 Table Primers used for PCR and sequencing of *vmpA* and *vmpB* loci. (DOCX)

S4 Table Codon-based Fisher’s exact test of selection between R1 repeat of VmpA types -I, II and III. Modified Nei-Gojobori method (Zhang et al. 1998); Trs/Trv = 2. P values less than 0.05 are highlighted in yellow.
(XLS)

S5 Table Codon-based Fisher’s exact test of selection between R1 VmpB (B) types -I, II and III. Modified Nei-Gojobori method (Zhang et al. 1998); Trs/Trv = 2. P values less than 0.05 are highlighted in yellow.
(XLS)
