# Supplementary figures and images for: When a Palearctic bacterium meets a Nearctic insect vector: Genetic and ecological insights into the emergence of the grapevine Flavescence dorée epidemics in Europe
Source: PLoS Pathog. 2020 Mar 25;16(3):e1007967. doi: 10.1371/journal.ppat.1007967 (PMC7135369; doi:10.1371/journal.ppat.1007967)

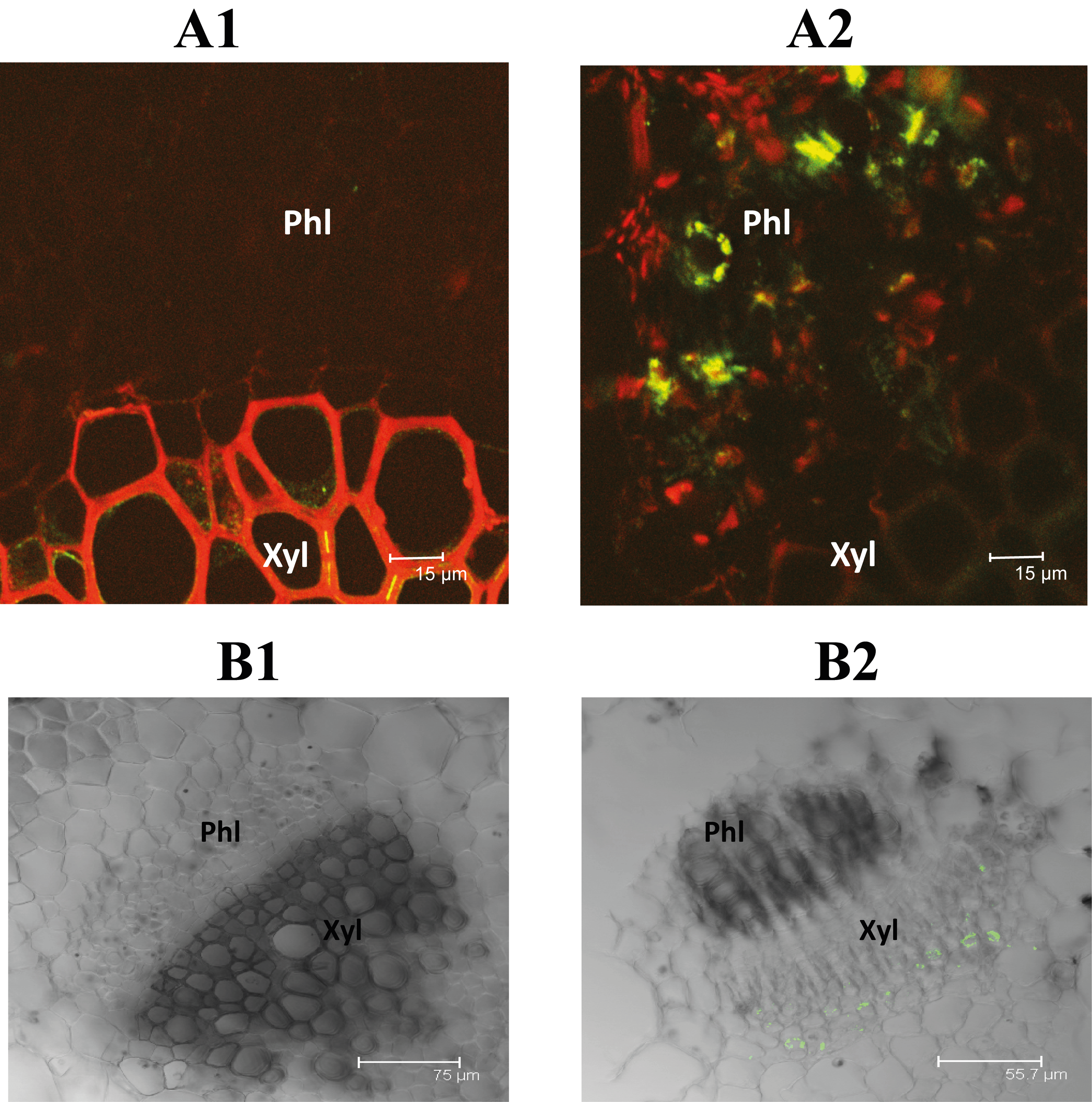

Supplement: S1 Fig — A: anti-VmpA monoclonal antibody detected with FITC labelled anti-mouse secondary antibodies in epifluorescent microcoscopy. B: anti-VmpB polyclonal antibodies detected with Alexa 488 labelled anti-mouse secondary antibodies in confocal microscopy. A1 and B1: healthy faba beans, A2 and B2: faba beans infected with FDP strain FD92. Xyl indicate xylem tissues and Phl indicate phloem tissues. (TIF) [file ppat.1007967.s001.tif]
