# Supplementary material for: When a Palearctic bacterium meets a Nearctic insect vector: Genetic and ecological insights into the emergence of the grapevine Flavescence dorée epidemics in Europe
Source: PLoS Pathog. 2020 Mar 25;16(3):e1007967. doi: 10.1371/journal.ppat.1007967 (PMC7135369; doi:10.1371/journal.ppat.1007967)
Supplement: S2 Table — (DOCX) [file ppat.1007967.s003.docx]

**S2 Table. Transmission trials of FD-related phytoplasmas by *O. alni*, *Allygus spp.* and *O. ishidae* collected on alder and subsequent transmission by *E. variegatus* and *S. titanus***

| **Insect gender species** | **Region** | **Nb of insects** | **% of infected insects** | **Nb of infected plants/nb inoculated^1^** | **Phytoplasma isolate** | ***Map* genotype*/ vmpA* cluster** | ***E. variegatus* / *S. titanus* transmission^2^** |
| --- | --- | --- | --- | --- | --- | --- | --- |
| ***Oncopsis alni*** | Palatinate/  Rhineland | 790 | 19 | 7/18 beans  0/7 alders | FG-09-2  FG-09-5  FG-09-6  FG-09-7  FG-09-9  FG-14-5  FG-15-101 | M52/I  M78/I  M52/I  M120/I  M43/I  M53/ nd  M14/I | -/nd  -/nd  -/nd  -/nd  -/nd  -/-  -/- |
|  | Alsace | 317 | 17 | 2/6 beans  0/7 alders | FF-10-8  FF-13-2 | M41/I  M46/I | -/-  -/- |
|  | Aquitaine | 142 | 17 | 3/3 beans  1/1 alder | FF-09-13  FF-10-2  FF-10-5  AF-15-29 | M45/I  M53/I  M21/I  M48/nd | -/nd  -/-  -/-  nd/nd |
|  | Burgundy | 109 | 45 | 1/2 beans  0/2 alders | FF-13-1 | M52/I | -/- |
| ***Allygus mixtus or modestus*** | Palatinate/  Rhineland | 178 | 66 | 2/9 beans  1/5 alders | FG-15-124  FG-15-125  AG-15-120 | M38/II  M38/II  M38/II | +/+  +/+  nd/nd |
|  | Alsace | 11 | 63 | 0/1 alder |  |  |  |
|  | Aquitaine | 28 | 36 | 0/1 bean |  |  |  |
|  | Burgundy | 32 | 44 | 0/3 beans  0/1 alder |  |  |  |
| ***Orientus ishidae*** | Palatinate/  Rhineland | 23 | 61 | 1/2 beans  1/1 alder | FG-15-126 AG15-115 | M38/II  M38/II | +/+  nd/nd |
|  | Alsace | 11 | 55 | 1/1 alder |  |  |  |
|  | Aquitaine | 104 | 49 | 5/5 alders | AF-15-31  AF-15-32  AF-15-33  AF-15-34  AF-15-38 | M50/nd  M50/III  M50-M38/nd  M50-M38/nd  M38/III | nd/nd  nd/nd  nd/nd  nd/nd |
|  | Burgundy | 16 | 56 | 0/3 beans  0/1 alder |  |  |  |

1. Transmission assay on one broad bean or alder plant
2. Acquisition on one infected broad bean plant followed by transmission on 5 to 10 broad bean plants.
