# Supplementary material for: When a Palearctic bacterium meets a Nearctic insect vector: Genetic and ecological insights into the emergence of the grapevine Flavescence dorée epidemics in Europe
Source: PLoS Pathog. 2020 Mar 25;16(3):e1007967. doi: 10.1371/journal.ppat.1007967 (PMC7135369; doi:10.1371/journal.ppat.1007967)
Supplement: S3 Table — (DOCX) [file ppat.1007967.s004.docx]

**S3 Table**. **Primers used for PCR and sequencing of *vmpA* and *vmpB* loci.**

| Gene name | Primer name | Primer sequence (5’-3’) | Method | PCR condition | Fragment lengths (bp)^1^ |
| --- | --- | --- | --- | --- | --- |
| *vmpA* | VMPA-F5 | CCTTATCAACTGGATATGGT | First PCR | 94°C – 5 min,  (94°C - 30 sec,  56°C – 30 sec,  66°C – 2 min) x 20 times,  66°C – 5 min | 2503  2269  2035 |
|  | VMPA-R3 | CTGATGCGTTTAGCCACTTC |  |  |  |
|  | VmpA-F8 | TTATAGAAATTATTCTCACAA | Second PCR | 94°C – 5 min,  (94°C - 30 sec,  48°C – 30 sec,  66°C – 2 min) x 35 times,  66°C – 5 min | 1956  1722  1488 |
|  | VmpA-R7 | TTTCATAACATTTATAATTATAC |  |  |  |
|  | VMPA-F3 | GATAGGAAAACAAAATGATAG | Sequencing |  | 1350  1116  882 |
|  | VMPA-R5 | AAAGCTAAAGATGCTACACC |  |  |  |
| *vmpB* | VmpB-F1 | GAATTTTGGTTCTTCAGCATTATTTTG | First PCR | 94°C – 5 min,  (94°C - 40 sec,  52°C – 40 sec,  66°C – 1 min 45 sec) x 40 times,  66°C – 5 min | 1703  1469  1235 |
|  | 31-11-D | CTAGAAATTTTTTGTTATTTCCG |  |  |  |
|  | 6282VMPB-F | GATAATAAAGAGGAATTGTTTTTGCG | Second PCR | 94°C – 5 min,  (94°C - 40 sec,  52°C – 40 sec,  66°C – 1 min 45 sec) x 40 times,  66°C – 5 min | 1208  974  740 |
|  | 31-11-DN | TAAGTATTATTATCATCCATAACC |  |  |  |
|  | 6282VMPB-F | GATAATAAAGAGGAATTGTTTTTGCG | Sequencing |  |  |
|  | VmpB-R2 | TAAGTATTATTATCATCCATAACC |  |  |  |

1. Length of the PCR fragments and of the theoretical sequenced fragments of the different 16SrV phytoplasma isolates (does not include a 3nt to 6 nt insertion-deletion depending on isolates).
